# Diurnal rhythms in gene expression in the prefrontal cortex in schizophrenia

**DOI:** 10.1038/s41467-019-11335-1

**Published:** 2019-08-09

**Authors:** Marianne L. Seney, Kelly Cahill, John F. Enwright, Ryan W. Logan, Zhiguang Huo, Wei Zong, George Tseng, Colleen A. McClung

**Affiliations:** 1 Translational Neuroscience Program, Department of Psychiatry, Center for Neuroscience, University of Pittsburgh, Pittsburgh, 15213 PA USA; 2 Department of Biostatistics, University of Pittsburgh, Pittsburgh, 15261 PA USA; 3 Department of Biostatistics, University of Florida, Gainesville, 32611 FL USA

**Keywords:** Circadian regulation, Schizophrenia, Gene expression

## Abstract

Schizophrenia is associated with disrupted cognitive control and sleep-wake cycles. Here we identify diurnal rhythms in gene expression in the human dorsolateral prefrontal cortex (dlPFC), in schizophrenia and control subjects. We find significant diurnal (24 h) rhythms in control subjects, however, most of these transcripts are not rhythmic in subjects with schizophrenia. Instead, subjects with schizophrenia have a different set of rhythmic transcripts. The top pathways identified in transcripts rhythmic only in subjects with schizophrenia are associated with mitochondrial function. Importantly, these rhythms drive differential expression patterns of these and several other genes that have long been implicated in schizophrenia (including *BDNF* and GABAergic-related transcripts). Indeed, differential expression of these transcripts is only seen in subjects that died during the night, with no change in subjects that died during the day. These data provide insights into a potential mechanism that underlies changes in gene expression in the dlPFC with schizophrenia.

## Abstract (web summary)

Sleep disturbance is common in psychiatric disease, and this may contribute to altered circadian rhythm in gene expression. Here the authors show that rhythms in gene expression in the dorsolateral prefrontal cortex in schizophrenia are different to that seen in healthy controls.

## Introduction

Schizophrenia is a psychiatric disease associated with positive (psychosis-related), negative (affect-related), and cognitive symptoms. One key feature of schizophrenia is disturbances in the sleep/wake cycle, including insomnia, poor sleep consolidation, and disrupted sleep architecture^1,2^. Moreover, prominent disruptions in diurnal rhythmicity of activity patterns, cortisol and melatonin profiles, and body temperature rhythms are also commonly associated with the disease. For example, Wulff et al.^3^ found that a subset of subjects with schizophrenia free run, similar to totally blind people with non-24 disorder, suggesting their rhythms are not entrained by the light/dark cycle, while others showed highly disorganized rest-activity rhythms. Moreover, a recent study by Johansson et al.^4^ measured circadian gene expression in blood samples and cultured fibroblasts from subjects with schizophrenia and found a loss of rhythmicity and decreased expression in core circadian genes compared to healthy controls. While circadian alterations are evident in the periphery and in behavior in subjects with schizophrenia, it is unclear whether molecular rhythms are altered in the brain, particularly in the dorsolateral prefrontal cortex (dlPFC), which is thought to be critical to the core symptomatology of the disease.

Several studies have identified differential gene expression in numerous transcripts in human cortical regions in subjects with schizophrenia, although these studies do not consider potential effects of circadian rhythms in gene expression. The most consistent results include decreased expression of genes associated with GABAergic transmission and mitochondrial function, and increased expression of genes associated with neuroimmune function^5–10^. However, most of these changes are modest in schizophrenia cohorts, can be inconsistent from study to study, and the mechanisms that lead to these changes remain unknown. A potential factor contributing to the variability in these studies is time-of death (TOD), which can be used to measure circadian pattern of gene expression.

Previously, we used a TOD analysis to measure molecular rhythms of gene expression in the human postmortem brain, with a focus on determining whether there was an effect of aging on these rhythms^11^. We identified a number of rhythmically-expressed genes with strong phase concordance across cortical regions. Moreover, the phase and amplitude of these rhythms was remarkably similar to those generated in a previous study by Li et al.^12^ using brains from a separate cohort and similar statistical algorithms, giving us a high degree of confidence in our measures. Here, we performed a similar time-of-death analysis using RNA-sequencing (RNA-seq) data generated by the CommonMind Consortium^13^ comparing healthy comparison (control) subjects to subjects with schizophrenia.

## Results

### Rhythmic gene expression in human dlPFC

**Fig. 1:**
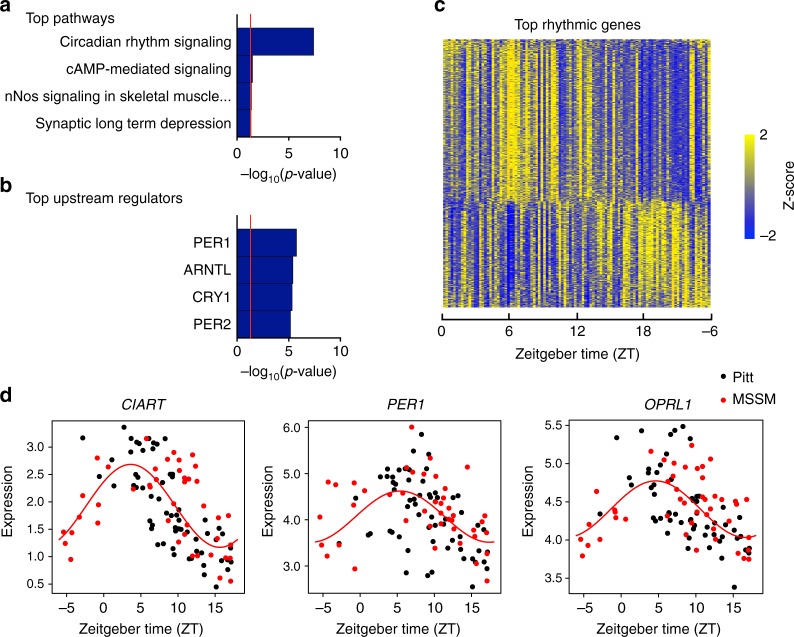
Circadian rhythms in gene expression in human DLPFC in healthy control subjects. Nonlinear regression was used to detect circadian gene expression patterns based on individual TODs. Sinusoidal curves were fitted using the nonlinear least-squares method and the coefficient of determination (R^2^) was used as a proxy of goodness-of-fit. A null distribution of R^2^ generated from 1,000 TOD-randomized expression data sets was used to estimate the empirical p-value by comparing observed R^2^ and the null distribution of R^2^. **a** The top pathway represented by these rhythmic genes is Circadian rhythm signaling. **b** The top predicted upstream regulators are core circadian genes. **c** Heatmap for circadian genes for all 104 healthy subjects (*p* < 0.01). Expression levels are Z-transformed for each gene, and the genes are ordered by their circadian phase value (peak hour). Each column represents a subject and the subjects are ordered by time of death. **d** Scatterplots representing rhythms in gene expression for the top 3 circadian genes in healthy controls. Each dot indicates a subject with *x*-axis indicating the time of death (TOD) on ZT scale (−6 to 18 h) and *y*-axis indicating gene expression level. Subjects from the Pitt brain bank are in black while subjects from the MSSM brain bank are in red. The red line is the fitted sinusoidal curve

We first probed for diurnal, 24 h, patterns of gene expression in the dlPFC (BA9/46) of a cohort of control subjects supplied by the University of Pittsburgh (Pitt) and Mt. Sinai School of Medicine (MSSM) (*N* = 104, Supplementary Data 1 and Supplementary Table 1) using RNA-seq. Nonlinear regression was used to detect circadian gene expression patterns based on individual TODs. Sinusoidal curves were fitted using the nonlinear least-squares method and the coefficient of determination (*R*^2^) was used as a proxy of goodness-of-fit. A null distribution of *R*^2^ generated from 1000 TOD-randomized expression data sets was used to estimate the empirical p-value by comparing observed R^2^ and the null distribution of *R*^2^. Indeed, we found that approximately 19% of detected genes are rhythmic (*p* < 0.05: 2685 genes; *p* < 0.01: 837 genes; Supplementary Data 2). The top pathway represented by these rhythmic genes is Circadian Rhythm Signaling (Fig. 1a), many rhythmic transcripts are known core circadian genes (e.g., *PER1*, *PER2*, *DBP*, *CIART*), and many of the rhythmic transcripts are genes with predicted circadian upstream regulators (e.g., *PER1*, *PER2*, *ARNTL*, *CRY1*; Fig. 1b). These results give us further confidence in our approach for measuring rhythmicity of gene expression using RNA seq data in the human postmortem brain. Of these rhythmic transcripts, we see that some peak during the day and some peak during the night (Fig. 1c; Supplementary Data 2), including transcripts with the most highly significant diurnal rhythms, *CIART*, *PER1*, and *OPRL1* (Fig. 1d). Notably, these findings are not driven by results from one brain bank, as there is a high degree of overlap between Pitt and MSSM data (Fig. 1d). Additionally, most of the top rhythmic genes were also identified as top rhythmic genes in our prior study^11^, and there was strong concordance in phase (88%) between the most highly rhythmic genes in BA9/46 and those found in BA47 from our previous study (Supplementary Figure 1). Together, these results indicate that diurnal gene expression rhythms exist in the human postmortem brain, and that many of these genes are similarly rhythmic across cortical brain regions.

### Rhythms are different in subjects with schizophrenia

Given evidence for disrupted circadian rhythms^1–4^ and dlPFC dysfunction in schizophrenia^14,15^, we next asked whether subjects with schizophrenia might have altered gene expression rhythms in dlPFC. From the CommonMind dataset, we identified 46 subjects with a schizophrenia diagnosis that met our strict criteria for inclusion in this analysis (i.e., known TOD within 2 h). We then selected 46 sex- and age-matched controls to create a cohort with the same sample size in each group (Supplementary Data 3). There were no significant differences in race, age, PMI, brain pH, brain collection site (Pitt/MSSM), or mean TOD between subject groups (Supplementary Table 2). P-values for the rhythmicity analysis were determined as for the full control cohort above.

**Fig. 2:**
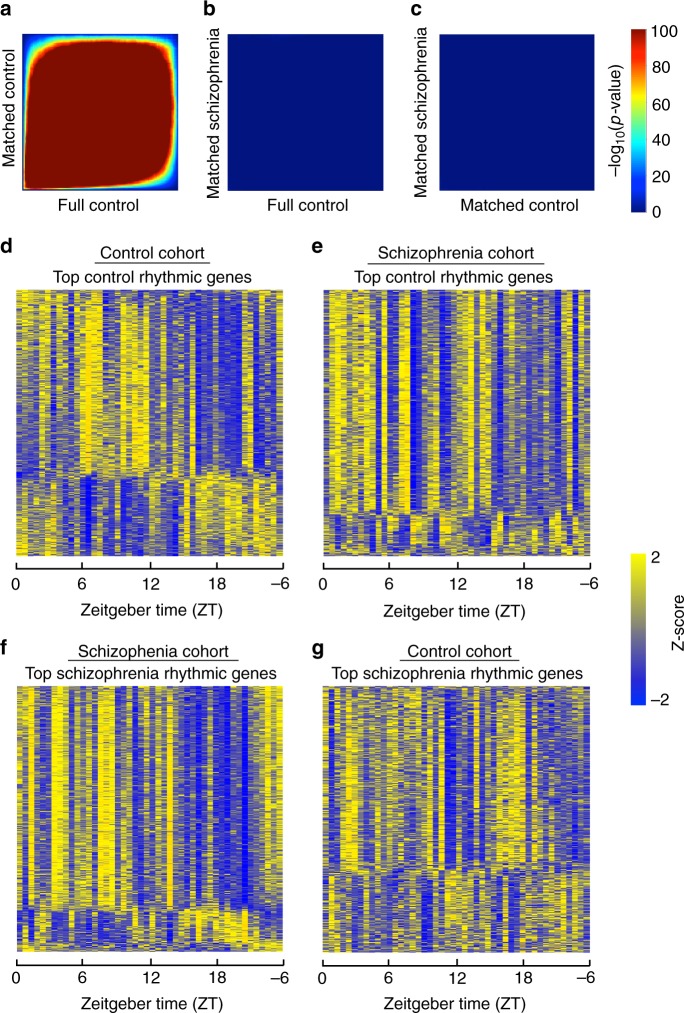
Rhythmic genes are largely distinct in control subjects and subjects with schizophrenia. Nonlinear regression was used to detect circadian gene expression patterns based on individual TODs. Sinusoidal curves were fitted using the nonlinear least-squares method and the coefficient of determination (*R*^2^) was used as a proxy of goodness-of-fit. A null distribution of *R*^2^ generated from 1000 TOD-randomized expression data sets was used to estimate the empirical p-value by comparing observed R^2^ and the null distribution of R^2^. **a** RRHO plot indicating high degree of overlap in rhythmic genes between the full healthy control cohort and the matched control cohort. **b** RRHO plot indicating lack of overlap in rhythmic genes between the full healthy control cohort and the matched schizophrenia cohort. **c** RRHO plot indicating lack of overlap in rhythmic genes between the matched healthy control cohort and the schizophrenia cohort. **d** Heatmap for the circadian genes in the matched control cohort (*p* < 0.05). Expression levels are Z-transformed for each gene, and the genes are ordered by their circadian phase value (peak hour). Each column represents a subject and the subjects are ordered by time of death. **e** Heatmap for the healthy control circadian genes in the schizophrenia cohort, indicating disrupted rhythmicity of normally rhythmic genes in subjects with schizophrenia. **f** Heatmap for the circadian genes in the matched schizophrenia cohort. **g** Heatmap for the schizophrenia circadian genes in the control cohort, indicating that these genes are not rhythmic in control subjects

**Fig. 3:**
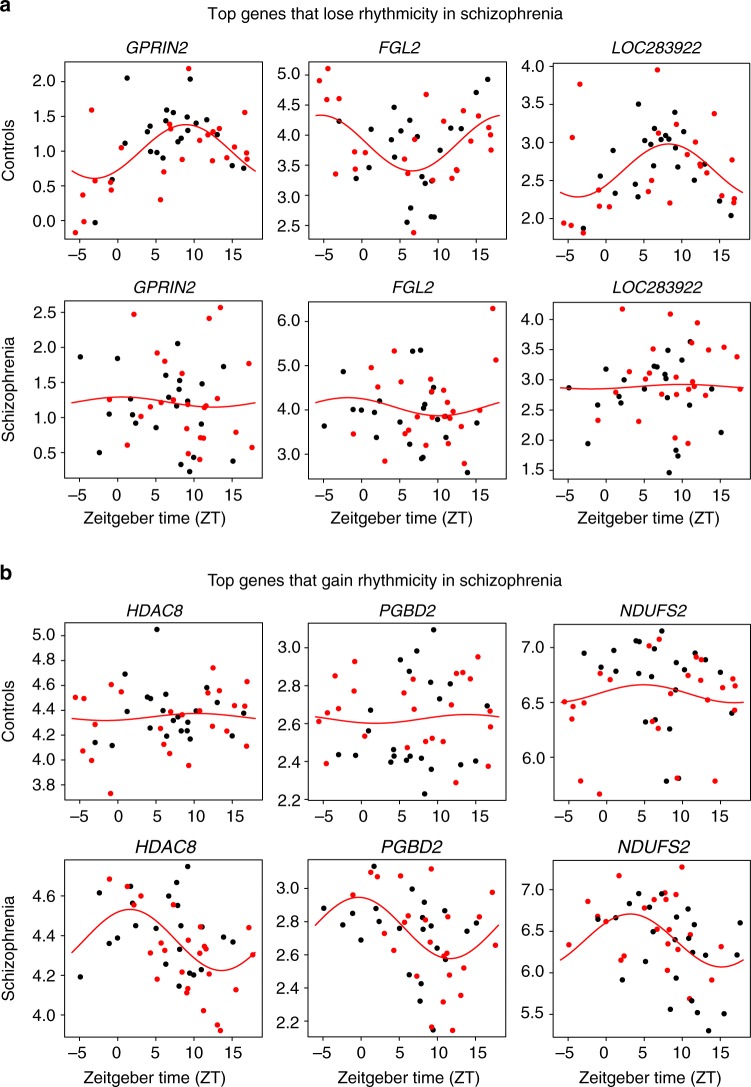
Scatterplots indicating rhythmicity for genes that lose or gain rhythmicity in subjects with schizophrenia compared to controls. Each dot indicates a subject with *x*-axis indicating the time of death (TOD) on ZT scale (−6 to 18 h) and *y*-axis indicating gene expression level. Subjects from the Pitt brain bank are in black while subjects from the MSSM brain bank are in red. The red line is the fitted sinusoidal curve. **a** Scatterplots indicating rhythmicity of *GPRIN2* (*p* < 0.0005), *FGL2* (*p* < 0.002), and *LOC283922* (*p* < 0.002) in healthy controls (top), which lose rhythmicity in subjects with schizophrenia (bottom). **b** Scatterplots indicating lack of rhythmicity of *HDAC8*, *PGBD2*, and *NDUFS2* in control subjects, but these genes gain rhythmicity in subjects with schizophrenia (*HDAC8*: *p* < 0.0005; *PGBD2*: *p* < 0.001; *NDUFS2*: *p* < 0.006).

We identified 707 diurnally rhythmic genes in control subjects and 708 rhythmic genes in subjects with schizophrenia at *p* < 0.05 (Supplementary Data 4). Notably, the diurnally rhythmic genes identified in each cohort were largely different, with an overlap of only 31 genes (Supplementary Data 5). The top 20 rhythmic genes in each group are listed in Supplementary Table 3. These genes that were rhythmic in both control and subjects with schizophrenia did not exhibit significant changes in phase, amplitude, or base, suggesting similar rhythmic patterns across groups (Supplementary Data 5). One possibility is that many of the genes that are rhythmic in control subjects just miss the cutoff for rhythmicity in subjects with schizophrenia, or vice versa. Thus, to more rigorously examine the overlap in rhythmic genes between subject groups, we employed Rank-Rank Hypergeometric Overlap (RRHO) analysis. RRHO is a threshold-free approach that examines the degree of overlap of identified genes across two data sets, which accounts for some genes just missing a threshold for significance in one group^16,17^. As expected, we found a very high degree of overlap in rhythmic genes between the full control cohort and the matched control cohort (Fig. 2a). Surprisingly, there was very little overlap of diurnally rhythmic genes identified in subjects with schizophrenia compared to either the full or matched control cohorts (Fig. 2b, c), suggesting completely distinct sets of genes are rhythmic in control and subjects with schizophrenia. Indeed, when assessing the statistical significance of the gene level findings in each group, there are very few genes that are significant in one group that just missed significance cutoff in the other group (Supplementary Figure 2). Notably, expression of the rhythmic genes in each subject group (Fig. 2d, f) did not show rhythmicity in the opposite subject group (Fig. 2e, g). The rhythmic genes in control subjects separate into 2 clusters; the top pathway for cluster 1 is circadian rhythm signaling, while the top pathways for cluster 2 relate to inflammation (Supplementary Figure 3). Interestingly, while the genes that are rhythmic in control subjects (Fig. 2d) do not have the same pattern of rhythmicity in subjects with schizophrenia, these genes may have a different temporal pattern of expression (e.g., 12-h rhythms) in schizophrenia (Fig. 2e). We next asked which genes lose or gain rhythmicity in subjects with schizophrenia compared to controls. Indeed, in schizophrenia, 424 transcripts lose rhythmicity, while 560 transcripts gain rhythmicity (Supplementary Data 6). Most of the rhythmic genes in one group fail to show rhythmicity in the other group, and vice versa (Fig. 3a, b). Taken together, these results suggest that subjects with schizophrenia have a very different set of diurnally rhythmic genes and a different rhythmic pattern in the dlPFC compared to control subjects.

### Rhythmicity of mitochondria-related genes in schizophrenia

**Fig. 4:**
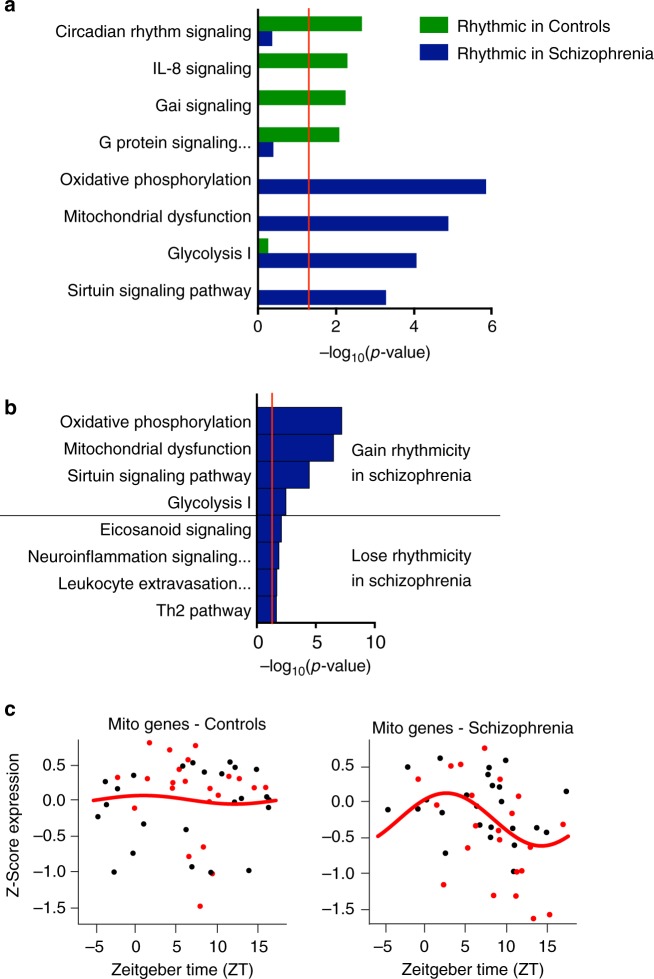
The top pathways represented by the rhythmic genes in control subjects and subjects with schizophrenia are completely distinct. While the top pathway in healthy controls relates to circadian rhythm signaling (**a**), the top pathways in subjects with schizophrenia relate to oxidative phosphorylation and mitochondrial dysfunction. **b** The top pathways for genes that gain rhythmicity in schizophrenia compared to healthy controls are oxidative phosphorylation and mitochondrial dysfunction. **c** Expression of mitochondrial-related genes in control (left) and subjects with schizophrenia (right). Expression for each gene was Z-transformed and averaged to create a Z-mitochondria (Z-mito) score for each subject. These Z-mito values are plotted across time of day. These mitochondrial-related genes are not rhythmic in healthy controls. In subjects with schizophrenia, mitochondrial-related genes peak between ZT 0–5

Having identified different patterns of rhythmic genes in control subjects and subjects with schizophrenia, we next asked if these genes are enriched for specific biological pathways. Similar to the full control cohort, the top pathway represented by the rhythmic genes in the matched control cohort was Circadian Rhythm Signaling; this pathway was not significant in the schizophrenia cohort, though multiple core circadian genes do maintain rhythmicity (*CIART* and *CRY1,* for example) but with a reduced amplitude (Supplementary Data 4, Fig. 4a). However, the top pathways represented by the schizophrenia rhythmic genes were oxidative phosphorylation and mitochondrial dysfunction (Fig. 4a). Furthermore, the top pathways for genes that gain rhythmicity in schizophrenia are also Oxidative Phosphorylation and Mitochondrial Dysfunction, suggesting that these pathways are indeed not rhythmic in control subjects, but become rhythmic in schizophrenia (Fig. 4b). On the other hand, the top pathways represented by genes that lose rhythmicity in schizophrenia are related to immune function (e.g., Eicosanoid Signaling, Neuroinflammation Signaling Pathway; Fig. 4b). The fact that genes related to oxidative phosphorylation and mitochondrial function gain rhythmicity in schizophrenia is particularly intriguing, as these same pathways are often identified as being disrupted in schizophrenia (reduced expression in schizophrenia compared to controls^9,10^).

We further investigated whether rhythmic changes in the expression of these genes might drive the differential expression findings in schizophrenia. In other words, might these oxidative phosphorylation- and mitochondrial function-related genes dip in expression during part of the 24-h day, with this dip in expression driving reduced expression findings. To test this hypothesis, we first examined diurnal patterns of expression of a set of 143 genes related to mitochondrial function (Supplementary Data 7). Consistent with the pathway analysis, we find that these mitochondrial function-related genes are, as a group, arrhythmic in control subjects, but have a strong diurnal rhythm in subjects with schizophrenia. Remarkably, in subjects with schizophrenia, these genes all appear to peak in the morning and trough during the evening (peak around ZT3-4, Fig. 4c). Additionally, in terms of overall expression levels, these transcripts largely match expression of control subjects during their peak (during the day) but dip below expression of control subjects during their trough (during the night).

### Differential expression is driven by rhythm changes

**Fig. 5:**
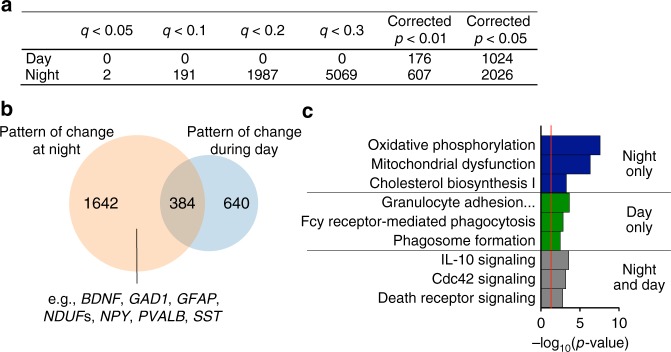
More genes are differentially expressed between schizophrenia and control subjects if they died during the night. **a** Table showing number of DE genes during day or night at various significance cutoffs. A permutation test was used to return corrected p-values and Storey’s *q* value was used to correct for multiple testing. **b** Venn diagram indicating overlap in genes that are DE at *p* < 0.05 in schizophrenia at night and during the day. Many genes that have previously been identified as being disrupted in schizophrenia are DE only at night (e.g., *BDNF*, *PVALB*, *SST*). **c** The pathways represented by the genes that are DE only at night are related to oxidative phosphorylation and mitochondrial dysfunction. The pathways represented by the genes that are trending towards DE only during the day relate to immune function. For the genes that are DE both during the day and night, the top pathways are related to immune function and Cdc42 signaling

**Fig. 6:**
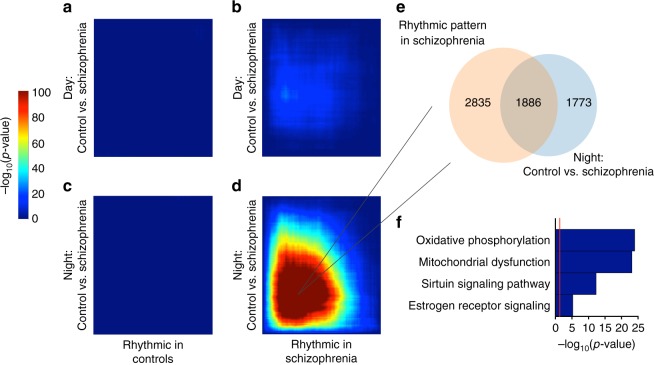
Several genes that are rhythmic in schizophrenia are also DE in subjects that died at night. **a**–**d** RRHO plots indicating that there is a high level of overlap between genes that are changed in schizophrenia during the night and rhythmic in subjects with schizophrenia. Venn diagram (**e**) indicating overlap in genes that are DE at night and rhythmic in schizophrenia and top pathways (**f**) for these genes relate to oxidative phosphorylation and mitochondrial dysfunction

To further test our hypothesis that certain previously identified differential expression findings are driven by rhythmicity differences in subjects with schizophrenia, we examined differential expression after splitting our cohort into day (ZT0-12) and night (ZT12-24) cohorts representing those that died during the day and those that died during the night. Remarkably, we find that when only analyzing subjects that died during the day, there are no genes that meet statistical criteria for differential expression (DE) between schizophrenia and control subjects, even at *q* < 0.3 (Fig. 5a and Supplementary Data 8; Storey’s *q* value correction). However, even though we had fewer subjects that died during the night compared with the day, we find several differentially expressed genes between subjects with schizophrenia and controls at *q* < 0.3 and below (Supplementary Data 9; Fig. 5a). Notably, many of the genes that are DE only in subjects that died at night are genes consistently implicated in schizophrenia in other DE studies (e.g., *BDNF*, *PVALB*, *SST*) which have not taken time of death into consideration (e.g.,^18–24^). Specifically, we compared our DE list with the meta-analysis DE data set generated recently by Gandal et al.^25^ and using RRHO found a high degree of overlap (Supplementary Figure 4). Moreover, the fold change in expression of these genes is much greater (many greater than 2 fold) when only examining subjects that died at night compared to other studies that do not take time of death into account^18,26^. While there were no genes that are DE in subjects that died during the day after correction for multiple testing, we performed an exploratory analysis to examine any potential overlap in patterns of gene expression changes between day and night DE genes. Here, we used a loose criterion with a corrected p-value of *p* < 0.05. Using this criterion, we find that most of those genes that are trending towards DE in subjects that died during the day are also DE in subjects that died at night (Fig. 5b; Fisher’s exact *p* < 10^–15^), suggesting that the levels of these genes are consistently up or down compared to controls regardless of the time of death. The pathways represented by genes that are DE only in subjects that died at night are primarily related to mitochondrial function, but also contain genes related to neurotransmission and GABAergic signaling (*GAD1, GAD2, NPY, PVALB, SST, CCK,* for example) which are mostly downregulated (Fig. 5c). Interestingly, those genes trending as DE only in subjects that died during the day, or in both day and night are primarily related to immune function and Cdc42 signaling (Fig. 5c). This suggests that DE of these particular transcripts are likely not driven by diurnal rhythms, but rather more constitutive factors, however it is worth noting that a number of other immune-related transcripts are also upregulated in schizophrenia only during the night. We next performed RRHO analysis to compare genes that were DE in subjects that died during the day or night with those that have diurnal rhythms in control subjects or subjects with schizophrenia. We found a large overlap only in genes that are DE in subjects that died during the night and genes that are rhythmic in subjects with schizophrenia (Fig. 6). We then examined the shared genes that are DE at night and rhythmic only in subjects with schizophrenia and found: 1) there is a very high degree of overlap between these two gene lists (Fig. 6d, e); 2) the top pathways represented by these genes are largely involved in oxidative phosphorylation and mitochondrial dysfunction (Fig. 6f). This suggests that the DE of these genes is driven by the fact that they have a diurnal pattern of expression in subjects with schizophrenia that is not present in control subjects, with factors specifically changing their expression during the night.

## Discussion

Our data demonstrates that subjects with schizophrenia have a set of genes that display a diurnal rhythm in the dlPFC, while also losing rhythmicity of genes that are normally rhythmic in control subjects. The genes that gain rhythmicity in subjects with schizophrenia are enriched for mitochondrial-related functions. Furthermore, genes that gain rhythmicity in schizophrenia appear to be the primary drivers of the differential-gene expression of many transcripts seen in studies where TOD is not taken into consideration. We acknowledge, however, that it is not possible to determine whether day/night variations in gene expression interact with the process of death, or whether these are a temporally precise representation of circadian gene expression in the living human brain.

There are several possibilities as to the mechanisms that underlie these differences. Core circadian proteins are primarily transcription factors that regulate clock-controlled gene expression. These circadian proteins, such as CLOCK and ARNTL, bind not only each other, but a host of other co-factors that regulate transcription. In fact, while the core molecular clock remains the same in most tissues, the genes that are rhythmically controlled by this loop are largely different, likely due to tissue specific co-factors^27,28^. Using cluster analysis, we identified two separate clusters of genes in control subjects associated with circadian rhythms (cluster 1) and inflammation (cluster 2), including many genes within canonical NFκB signaling pathways, which could be integral to molecular clock function^29^. There are also indications that CLOCK can bind to factors like NFκB in situations where neuroinflammation occurs, resulting in increased regulation of a different set of CLOCK-controlled genes^30^. Our data indicates that there could be a consistent increase in neuroinflammation-related genes in schizophrenia across the day/night cycle. Thus, this high inflammatory state could drive the binding of core circadian proteins to a different set of co-factors (such as NFκB) in the dlPFC of subjects with schizophrenia, resulting in different rhythmic transcripts. Another possibility is that there are differences in the diurnal pattern of RNA degradation between schizophrenia and control subjects. It is becoming increasingly clear that processes impacting RNA stability, such as polyadenylation, have diurnal rhythms. For example, polyA tails of specific target mRNAs are degraded at particular times of day^31^. Certain transcripts might be directed towards degradation, particularly at night, in subjects with schizophrenia using a process that is different than the normal pathway in control subjects.

It is also possible that these rhythmic changes in mitochondrial function reflect diurnal differences in neuronal activity in the dlPFC of subjects with schizophrenia. Several studies have suggested that subjects with schizophrenia have deficits in excitatory/inhibitory balance within the dlPFC, with potential decreases in both pyramidal cell and interneuron activity^15^. What is less known is whether these deficits might be more pronounced during the night. One study by Hufford et al.^32^ found that subjects with schizophrenia had the highest level of cognitive function between 8–10am as measured by the MATRICS Consensus Cognitive Battery (MCCB), with cognitive function declining through the evening. Moreover, EEG activity studies during sleep find that subjects with schizophrenia have lower delta activity, fewer sleep spindles, and distinct differences in alpha, beta and theta power in frontal and occipital regions, which may reflect brain dysfunction during the night and often correlate with cognitive performance during the day^33–35^. The PFC is thought to be important in generating synchronized slow wave sleep activity. Thus, the frontal lobe dysfunction described in schizophrenia may be associated with the decreased EEG slow wave activity seen during sleep. It is interesting to note the phenomena of sundowning in people with dementia is quite common, with increased agitation, delusions, confusion, and psychotic symptoms only during the evening hours, which suggests diurnal regulation over the circuits in the brain that regulate these processes^36,37^. Interestingly, a circuit in the brains of mice has recently been reported which controls circadian rhythms in aggressive behavior^38^, and similar mechanisms could be involved in a change in brain function specifically at night.

Another possibility is the potential impact of medications on rhythms in gene expression. Many people take antipsychotic medications only at night before bed since they can be sedating^39^. The potential diurnal effects of these medications would depend on the half-life of the particular medication, which can vary tremendously based on how often they are taken. For example, Quetiapine has a half-life of 6–8 h and is typically taken twice daily, while olanzapine has a half-life of 21–54 h and is taken once daily^40,41^. Olanzapine pamoate has a half-life for elimination of 30 days and can be given at 2–4 week intervals^42^, thus it is unlikely that newer, longer lasting, formulations would impact daily rhythms in gene expression, however older medications could have more of an impact depending upon when they are taken during the 24 h period. For the subjects in our study, we do not have enough information regarding their medication habits to be able to determine if this is a potential mechanism. However, it is worth noting that other studies have looked at the effects of chronic antipsychotic treatment on gene expression in the PFC of non-human primates including some of the genes identified as DE at night in this study (e.g., *BDNF*, *OAT*) and have found no differences in expression as a result of antipsychotic treatment^9,24^.

One potential limitation to our study is the relatively small sample size that might drive our differential expression findings. Related to this possibility, Fromer et al., reported fewer DE genes in schizophrenia than other studies with fewer subjects. They then used simulated data to show that smaller sample sizes will result in a higher probability of false positives^13^. One possible reason why smaller sample size might result in more false positives is due to cell type heterogeneity. Different cell types within a bulk tissue sample can contribute different patterns of gene expression. Therefore, as group sample sizes get smaller, there may be false positives in terms of increasing numbers of DE genes based solely on random sampling of cell types. However, our results for genes that are DE at night are highly consistent with results from other postmortem brain gene expression studies in schizophrenia^5–10,25^, increasing our confidence in our results. Here, we are accounting for a variable (TOD), which we show has a large effect on the overall variance in the data. We would argue that the collection of studies so far that have used these rhythm analyses in human postmortem brain tissue suggest that TOD differences between subjects is a major driver of the overall variance in gene expression. Therefore, even though we have fewer subjects when we split by day/night, we are enriching for the detectability of the signal. As an example, numerous studies have shown reductions in mitochondrial-related genes in schizophrenia, with fairly modest effect sizes. We show that by accounting for TOD, the signal is enhanced in subjects that died at night, with little to no changes during the day; ignoring time of day essentially dilutes the signal. This highlights the utility of examining disease by time of day comparisons.

In conclusion, we show that we can identify rhythmic transcripts in the human brain using RNA-sequencing data and a TOD analysis, similar to previous approaches using targeted gene expression or microarrays^11,12,43^. We also find that rhythms in the dlPFC of subjects with schizophrenia are profoundly different from those detected in control subjects. Many of the identified rhythmic transcripts are involved in mitochondrial function and have a peak during the day and trough at night driving differential expression. Many other genes commonly found to have differential expression in schizophrenia, such as those involved in GABAergic transmission, are only altered in subjects that died at night, again suggesting that these rhythms may drive differential expression. It will be interesting in future studies to use additional analytical approaches, for instance to determine whether there are non-24 h rhythms or whether specific subject covariates influence rhythmic expression. Future studies will identify the mechanisms by which these changes are occurring and how they relate to disease symptoms.

## Methods

### Human postmortem brain samples

Human postmortem RNA-sequencing data for 613 subjects were obtained from the CommonMind Consortium (CMC)^13^ (https://www.nimhgenetics.org/available_data/commonmind/). Subjects were collected by the University of Pittsburgh and by Mt. Sinai School of Medicine. We used subjects meeting 3 criteria: 1) subjects with known time of death (TOD) and meeting the criteria of rapid death (<2 h elapsed time between precipitating event and death pronouncement); 2) subjects with age less than 65 years; 3) subjects with postmortem interval (PMI) less than 30 h. A total of 150 subjects fit these criteria, including 104 healthy control subjects and 46 subjects with schizophrenia^13^. TOD information on these subjects is included in Supplementary Data 1 and 4.

### Time of death analysis in the zeitgeber time scale

The TOD for each subject was collected at local time. Adjusting the local time to internal biological clock time, TODs were normalized to zeitgeber time (ZT) scale. TOD was first converted to coordinated universal time (UTC) by adjusting time zone and daylight savings time. An internal biological clock time – sunrise time was obtained by adjusting UTC with longitude, latitude and elevation of the death place. Each subject’s TOD was set as ZT = *t* h after previous sunrise (if 0 < *t* < 18) or before next sunrise (if 0 > *t* ≥ −6). The inferred ZT-scaled TODs can also be found in Supplementary Data 1.

### Matched cohort of control and schizophrenia subjects

The larger sample size (*n* = 104) for healthy control subjects may result in more statistical power for rhythm detection than the smaller sample size (*n* = 46) for subjects with schizophrenia. Therefore, we selected 46 healthy subjects best matched to the 46 schizophrenia subjects by age, sex, race, TOD, PMI, site of collection and pH. Detailed information about the matched cohort is in Supplementary Table 2.

### RNA-sequencing data preprocessing

All samples were analyzed using RNA-sequencing technology. A total of 30,714 unique genes were identified, which were log2 normalized. Genes were retained for analysis if counts per million (cpm) was greater than 1 in 50% or more subjects. All Y-chromosome genes were also eliminated along with transcripts with no identifiers. After filtering, 13,914 genes remained. Since samples in the CommonMind dataset were generated in 2 brain banks, site correction of normalized and filtered data was performed using the ComBat function of the SVA R package^44^. Additionally, we included equal proportions of Pittsburgh and Mt. Sinai individuals in each experimental group.

### Rhythmicity analyses

To detect circadian patterns of gene expression, we chose to use sinusoidal analysis for several reasons. First, this is the same type of analysis previously used in human postmortem brain circadian analysis (by our group and others)^11,12,43^. Second, other methods (e.g., JTK Cycle) are non-parametric tests that works best with large sample size and evenly spaced data points (i.e., regular time intervals), which is more commonly seen in animal studies or cell cycle; this even spacing is not possible using TOD analysis in human postmortem brain samples. Furthermore, our method assumes a parametric model. The benefit to this is that we can easily measure the rhythmicity, amplitude, phase, and peak parameters and compare these parameter values between genes and across subject groups. To consider covariates in the analyses, subject groups were matched across age, sex, race, TOD, PMI, site of collection, and pH, consistent with previous approaches using TOD analysis of gene expression in human postmortem brains^11,12,43^.

To assess the temporal rhythmicity of gene expression, sinusoidal curve regression was fit to each gene and TODs:

$$y = A{\rm{sin}}\left( {ft + p} \right) + b,$$

where *y* is the gene expression level, *A* is an amplitude factor, *f* = *π*/12 is fixed frequency such that 24-hours is a period, *t* is time to death, *p* is a phase factor and *b* is the offset. Levenberg-Marquardt algorithm was applied to solve the sinusoidal curve fitting problem, using nls.lm function of R package minpack.lm. In addition to the coefficient estimate *A*, *p* and *b*, peak hour of the circadian pattern and goodness-of-fit coefficient *R*^2^ were also calculated. Here *R*^2^ = 1 − *RSS*_m_/*RSS*_0_, where *RSS*_m_ is the residual sum of square of the fitted model and *RSS*_0_ is the residual sum of square of the null model (intersect only model). The null hypothesis is that there is no rhythmic pattern and *R*^2^ was used to assess the significance level. The null distribution of *R*^2^ was obtained by pooling *R*^2^ of all genes through fitting sinusoidal curve to shuffled data, where 1000 shuffled data were generated by randomly shuffling TOD. *P* values were obtained by comparing observed *R*^2^ and the null distribution of *R*^2^. False discovery rate was calculated by Benjamini-Hochberg procedure.

Subjects with schizophrenia (*n* = 46) and their matched comparison subjects (*n* = 46) were analyzed independently to detect rhythmicity patterns. The effects of schizophrenia on the rhythmicity pattern were assessed through permutation. The null hypothesis is that rhythmicity patterns of subjects with schizophrenia and comparison subjects are the same. Under the null hypotheses, we randomly shuffled the schizophrenia/comparison labels to generate an empirical null distribution.

Analyses were used to detect genes that lost or gained rhythmicity in subjects with schizophrenia. Genes with either a loss or gain in rhythmicity between control and subjects with schizophrenia were determined using the difference in *R*^2^ between the two cohorts (*ΔR*^*2*^ = $R_{{\rm{scz}}}^2 - R_{{\rm{ctrl}}}^2$). A loss of rhythmicity is defined as a gene that is more rhythmic in control than schizophrenia (*ΔR*^2^ < 0), while a gain in rhythmicity is defined as a gene that is more rhythmic in schizophrenia than control (*ΔR*^2^ > 0). Under the null hypothesis, there is no difference in rhythmicity between schizophrenia and controls (*ΔR*^*2*^ *=* *0*). To rigorously test the hypothesis, we generate a null distribution of *ΔR*^2^ using the null *R*^2^ values permuted in the schizophrenia and control cohorts separately. Any gene that showed significant *R*^2^ decrease or increase (*p* < 0.05 through permutation test) in schizophrenia would be annotated as loss of rhythmicity or gain of rhythmicity, respectively. For the loss of rhythmicity analysis, we restricted to genes that were significantly rhythmic in controls (*p* < 0.05) and exhibit a significant loss in rhythmicity in schizophrenia compared to controls (*p* < 0.05). For the gain of rhythmicity analysis, we restricted to genes that were significantly rhythmic in schizophrenia (*p* < 0.05) and exhibit a significant gain in rhythmicity in schizophrenia compared to controls (*p* < 0.05). We also assessed whether there were differences in phase, amplitude, or base between control and schizophrenia cohorts; we restricted analyses to genes that were significantly rhythmic in both control and subjects with schizophrenia for this analysis.

Expression levels are Z-transformed for each gene, and the genes are ordered by their circadian phase value (peak hour). Each column represents a subject and the subjects are ordered by time of death. A heatmap for the genes exhibiting circadian rhythms was generated for the full control cohort (104 subjects; *p* < 0.01). We also generated heatmaps for (1) the circadian genes in the matched control cohort (*p* < 0.05); (2) the circadian genes identified in the control cohort, but plotted for subjects with schizophrenia; (3) the circadian genes identified in the schizophrenia cohort (*p* < 0.05); (4) the circadian genes identified in the schizophrenia cohort, but plotted for matched control subjects. For matched control subjects, k means clustering was used to identify patterns of rhythmic genes.

We generated scatter plots representing rhythms in gene expression. Each dot indicates a subject with *x*-axis indicating the TOD on ZT scale and *y*-axis indicating gene expression level. The red line is the fitted sinusoidal curve. Scatter plots were generated for: (1) top 3 circadian genes identified in the full control cohort (104) subjects; (2) 3 genes that lose rhythmicity in subjects with schizophrenia compared to matched controls; and (3) three genes that gain rhythmicity in subjects with schizophrenia compared to matched controls.

A list of mitochondrial-related genes from INGENUITY pathway analysis (IPA) software (Qiagen) was used. After filtering out genes that were not expressed above background levels in our data set, 143 mitochondrial-related genes remained (Supplementary Data 7). We calculated a Z-score of mitochondrial-related gene expression for each subject. The Z-mitochondrial value for each subject was then plotted across TOD.

Coherence between the two studies (Chen et al. and the current study) is visualized in a phase concordance plot. The phases of the top 25 genes that are rhythmic in both studies were plotted against each other (Supplementary Figure 1). An overall measure of phase concordance between the two studies was calculated as the proportion of genes with peak difference less than or equal to 5 or greater than or equal to 20 to the total number of top genes.

### Pathway enrichment and upstream regulator analysis

INGENUITY pathway analysis (IPA) software (Qiagen) was used to identify molecular pathways enriched for and potential upstream modulators of identified gene lists. In the pathway analysis, gene lists were analyzed as follows: (1) the 13,910 annotated genes remaining after filtering were used as the background gene set; and (2) IPA pathways with < 15 or > 300 genes were not included. For the upstream regulator analysis, the input genes in the pathway analysis were used.

### Rank-rank hypergeometric overlap (RRHO) analysis

RRHO identifies overlap between two genes lists ranked by the −log_10_(*p* value)^16,17^ and avoids an arbitrary threshold in conventional Venn diagram approaches. Here, *p* values for all genes are used, not only genes that reach a threshold of significance. We used RRHO: (1) to identify the overlap in significantly rhythmic genes between the full control, matched control, and matched schizophrenia cohorts; (2) to assess the level of overlap between genes that were rhythmic in either the matched control or schizophrenia cohort with genes identified as differentially expressed (DE) during the day or at night; (3) to assess overlap between genes that we identified to be DE at night with genes previously reported to be DE in schizophrenia^25^.

### Differential expression analysis

We split the matched cohort into subjects that died either during the day (ZT0-12; *N* = 60 subjects) or during the night (ZT12-24; *N* = 32 subjects). We performed differential expression analysis on the disease effect for subjects that died during the day or night separately. Due to the nature of human postmortem tissue, many covariates are present in our data (PMI, RIN, medication use, sex) yet the sample size is relatively small. Because of this, we performed variable selection for a maximum of two covariates (not including disease effect) based on Bayesian information criterion (BIC) for each gene individually^45^. Supplementary Data 10 (day DE) and Supplementary Data 11 (night DE) indicate percent variance explained by covariates for DE genes. A boxplot illustrating the percent variance for these covariates for the night DE is shown in Supplementary Figure 5. For each gene, the percentage of variance for each covariate is calculated using the variance explained by each covariate divided by the total variance of the gene expression. The *p* value returned from the feature selection is biased due to the model differences between each gene. Thus, a permutation test is used to return a corrected empirical *p* value. We further corrected for multiple comparisons using Storey’s *q* value correction in the R package ‘*q* value’^46^

### Reporting Summary

Further information on research design is available in the Nature Research Reporting Summary linked to this article.

## Supplementary information

Supplementary Information

Description of Additional Supplementary Files

Supplementary Data 1

Supplementary Data 2

Supplementary Data 3

Supplementary Data 4

Supplementary Data 5

Supplementary Data 6

Supplementary Data 7

Supplementary Data 8

Supplementary Data 9

Supplementary Data 10

Supplementary Data 11

Reporting Summary

## Data Availability

The RNA sequencing data used in this analysis is available through the Common Mind Consortium through an approval process (https://www.nimhgenetics.org/available_data/commonmind/).

## Code availability

The code used in this analysis and instructions are available at GitHub (https://github.com/KellyCahill/Circadian-Analysis-).
