# Supplementary material for: Diurnal rhythms in gene expression in the prefrontal cortex in schizophrenia
Source: Nat Commun. 2019 Aug 9;10:3355. doi: 10.1038/s41467-019-11335-1 (PMC6689017; doi:10.1038/s41467-019-11335-1)
Supplement: Supplementary file 1 — Supplementary Information [file 41467_2019_11335_MOESM1_ESM.pdf]

Supplementary Information

Diurnal Rhythms in the Prefrontal Cortex in Schizophrenia Drive Differential Gene Expression

Seney et al.

Supplementary Tables

Supplementary Table 1. Description of the full control cohort.

	**Controls (n = 104)**
**Sex, No. (%)**	
Male	81 (78)
Female	23 (22)
**Race, No. (%)**	
White/Hispanic	86 (83)
Black	17 (16)
Asian	1 (1)
**Age, mean ± SD, y**	48.4 ± 12.3
**PMI, mean ± SC, h**	17.9 ± 6.0
**Brain pH, mean ± SD**	6.7 ± 0.2
**TOD, mean ± SD**	8.6 ± 5.7
**Site, No. (%)**	
Pitt	61 (59)
MSSM	43 (41)

Abbreviations: h, hours; PMI, postmortem interval; SD, standard deviation; TOD, time of death; y, years

Supplementary Table 2. Description of the matched cohort.

	**Control (n = 46)**	**Schizophrenia (n = 46)**
**Sex, No. (%)**		
Male	33 (72)	32 (70)
Female	13 (28)	14 (30)
**Race, No. (%)**		
White/Hispanic	35 (76)	34 (74)
Black	11 (24)	12 (26)
**Age, mean ± SD, y**	49.1 ± 13.2	50.1 ± 11.5
**PMI, mean ± SD, h**	17.3 ± 6.2	17.0 ± 8.3
**Brain pH, mean ± SD**	6.6 ± 0.2	6.5 ± 0.3
**TOD, mean ± SD**	7.0 ± 6.4	7.8 ± 5.2
**Site, No. (%)**		
Pitt	22 (48)	22 (48)
MSSM	24 (52)	24 (52)

Abbreviations: h, hours; PMI, postmortem interval; SD, standard deviation; TOD, time of death; y, years

Supplementary Table 3. Top 20 rhythmic genes in controls and schizophrenia.

**Control Top rhythmic**	**Schizophrenia Top rhythmic**
CIART	***CIART***
OPRL1	*WNT10B*
GPRIN2	*LAMB3*
PER2	***OPRL1***
ZFHX4-AS1	*CYB561*
IFRD2	*HDAC8*
FGL2	***NIM1K***
DNAH9	*DUBR*
LOC101929653	*KRT17P1*
LOC283922	*EBP*
ZNF844	*PGBD2*
NIM1K	*CTSK*
CRY2	*ZBTB22*
CAT	*NPRL2*
SLC6A6	*IFT122*
SELENOP	*NFATC4*
ANKDD1A	*RNF112*
ASB1	*VOPP1*
SAMD9	*NIT1*
ZNF251	*USF1*

Genes in bold are rhythmic in both controls and schizophrenia.

Supplementary Figures

Supplementary Figure 1. There is strong concordance in phase (88%) between the most highly rhythmic genes in BA46 and those found in BA47 from our previous study.

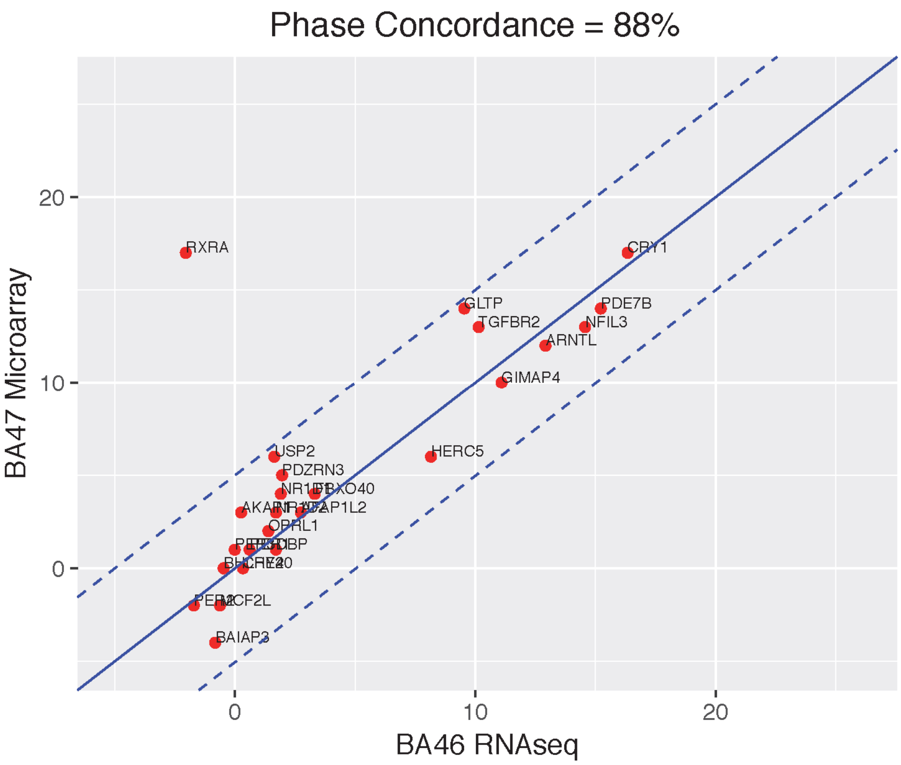

Supplementary Figure 2. **The scatter plot illustrates the relationship between significantly rhythmic genes in control subjects and subjects with schizophrenia across varying p-values.** The orange and yellow sections of the scatter plot illustrate genes that are highly significant (log10 pvalue > 1.3) in either schizophrenic or controls, and almost significant (0.6 < log10 pvalue < 1.3) in the opposite group. The majority of genes (in green) are only significant in one group and are not close to significance in the other.

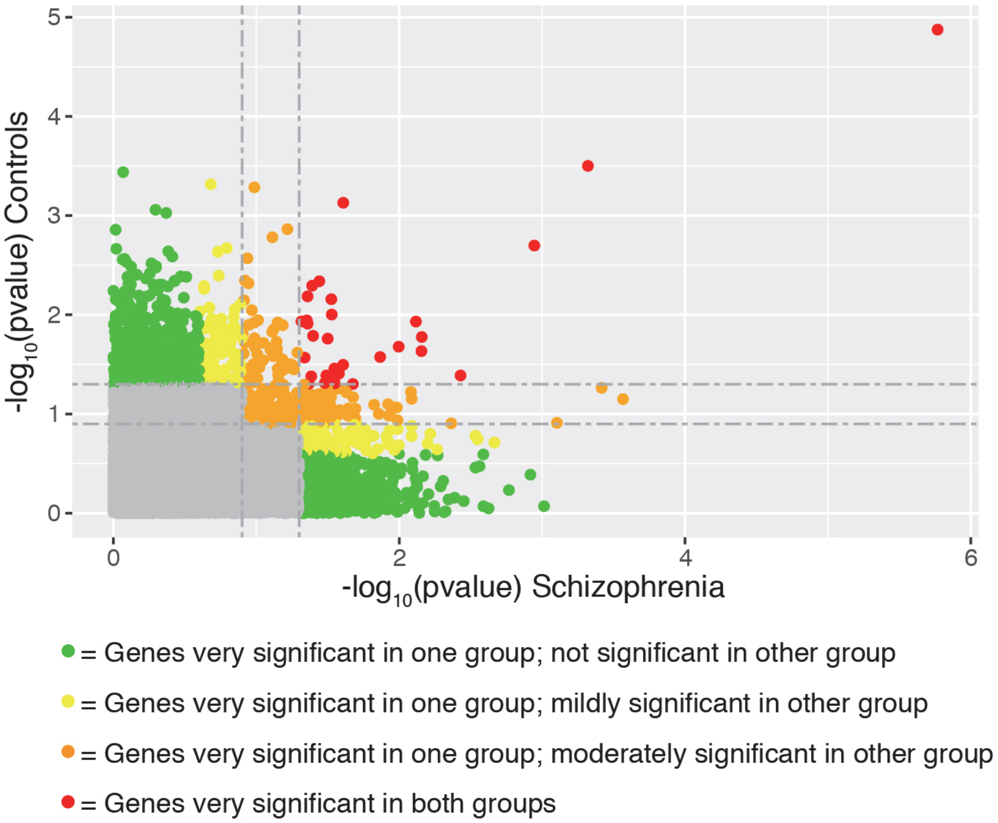

Supplementary Figure 3. Pathways represented by rhythmic genes in the matched control cohort. These rhythmic genes separate into 2 clusters. The top pathway represented by cluster 1 is circadian rhythm signaling, while the top pathways in cluster 2 relate to inflammation.

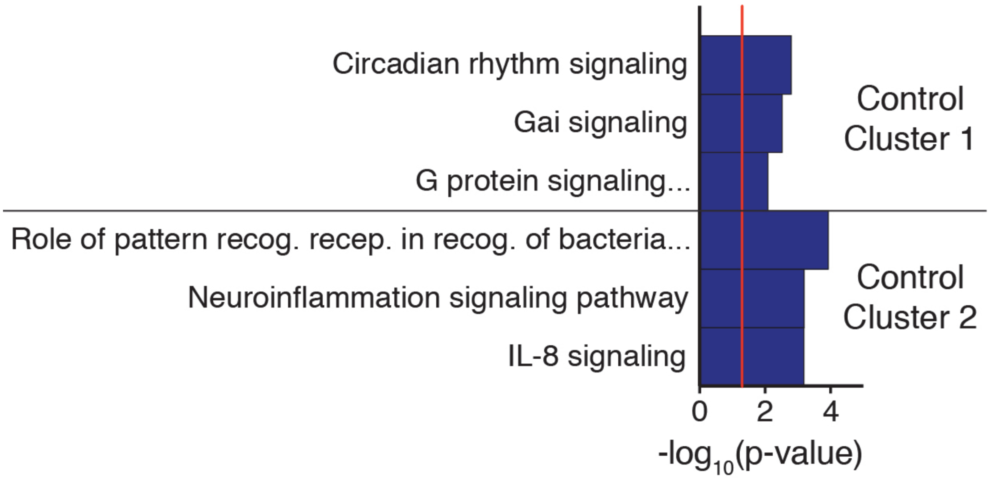

Supplementary Figure 4. High level of overlap between genes identified as being DE at night and previously identified DE genes in schizophrenia. The interpretation of the rank-rank hypergeometric overlap (RRHO) plot is shown on the left. A hot spot in the bottom left corner indicates overlap in genes upregulated in schizophrenia in both datasets. A hot spot in the top right corner indicates overlap in genes downregulated in schizophrenia in both datasets. A hot spot in the top left indicates overlap in genes upregulated at night in schizophrenia and downregulated in schizophrenia in the meta-analysis dataset from Gandal et al.,[1]. A hot spot in the bottom right indicates overlap in genes downregulated in in schizophrenia at night and upregulated in the Gandal et al., dataset. The RRHO plot on the right indicates that there is a high degree of overlap in genes upregulated in both datasets as well as a high degree of overlap in genes downregulated in both datasets.

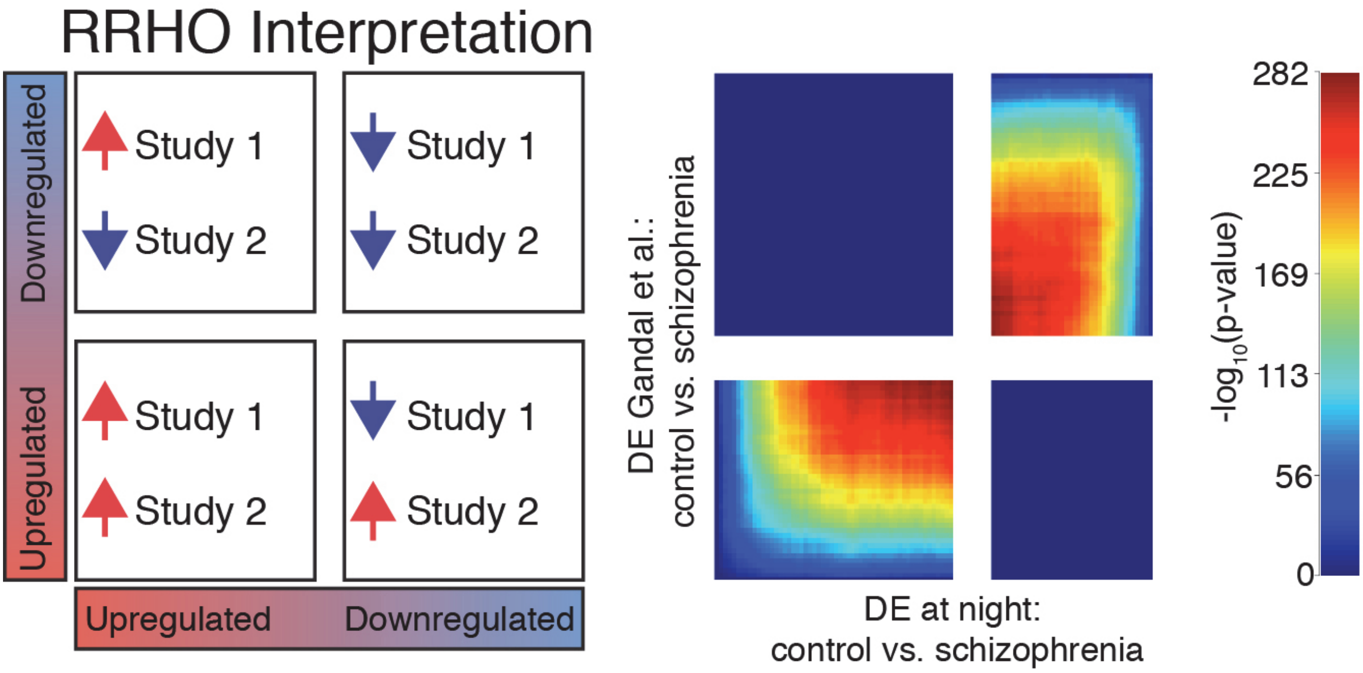

Supplementary Figure 5. Variation in DE analysis explained by each covariate. Boxplots of percentage of variation explained by each covariate (disease status, age, gender and PMI) in the 607 identified DE genes in subjects that died during the night. For each DE gene, 0, 1 or 2 confounding covariates among age, gender and PMI are selected. The boxplot for each covariate is drawn only for selected genes (number of selected genes of each covariate is shown).

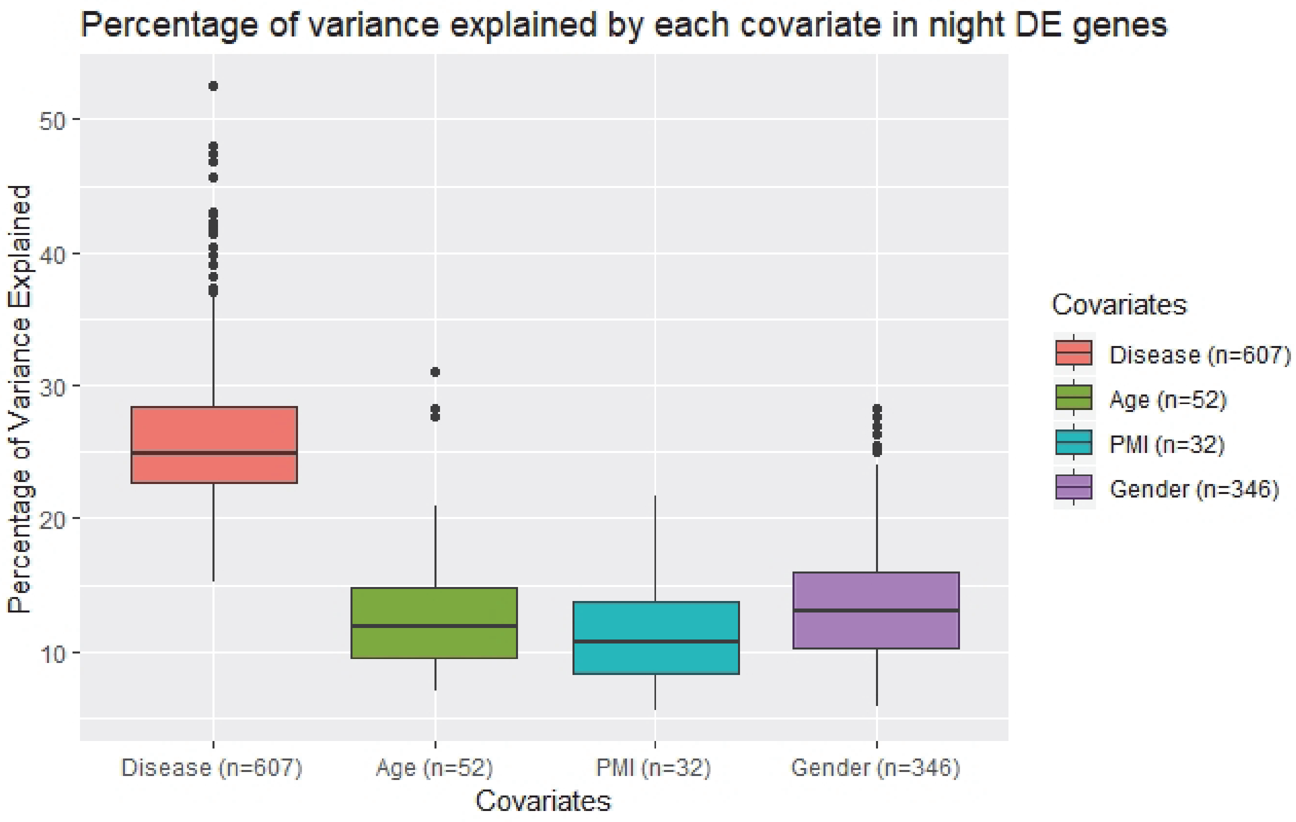

Supplementary References

1. Gandal, M.J. *et al.* Shared molecular neuropathology across major psychiatric disorders parallels polygenic overlap. *Science* **359**, 693-697 (2018).
