# Supplementary material for: Diurnal rhythms in gene expression in the prefrontal cortex in schizophrenia
Source: Nat Commun. 2019 Aug 9;10:3355. doi: 10.1038/s41467-019-11335-1 (PMC6689017; doi:10.1038/s41467-019-11335-1)
Supplement: Supplementary file 2 — Description of Additional Supplementary Files [file 41467_2019_11335_MOESM2_ESM.pdf]

Description of Additional Supplementary Files

File Name: Supplementary Data 1
Description: Cohort of control subjects from University of Pittsburgh and Mt. Sinai School of Medicine.

File Name: Supplementary Data 2
Description: Rhythmic genes in the dlPFC from human postmortem brains.

File Name: Supplementary Data 3
Description: Matched cohort between control subjects and subjects with schizophrenia for rhythmic analyses.

File Name: Supplementary Data 4
Description: Rhythmic genes in dlPFC in control subjects and subjects with schizophrenia.

File Name: Supplementary Data 5
Description: Overlap of rhythmic genes between control subjects and subjects with schizophrenia.

File Name: Supplementary Data 6
Description: Genes which lost or gained rhythmicity between control subjects and subjects with schizophrenia.

File Name: Supplementary Data 7
Description: Mitochondrial related genes.

File Name: Supplementary Data 8
Description: Comparison of genes between control subjects and subjects with schizophrenia who died during the day.

File Name: Supplementary Data 9
Description: Comparison of genes between control subjects and subjects with schizophrenia who died during the night.

File Name: Supplementary Data 10
Description: Differentially expressed genes during the day ($P<0.05$) with variance shown for each covariate.

File Name: Supplementary Data 11
Description: Differentially expressed genes during the night ($P<0.05$) with variance shown for each covariate.
