# Supplementary material for: Diurnal rhythms in gene expression in the prefrontal cortex in schizophrenia
Source: Nat Commun. 2019 Aug 9;10:3355. doi: 10.1038/s41467-019-11335-1 (PMC6689017; doi:10.1038/s41467-019-11335-1)
Supplement: Supplementary file 14 — Reporting Summary [file 41467_2019_11335_MOESM14_ESM.pdf]

nature research

Corresponding author(s): DBPR NCOMMS-19-00691-T

Last updated by author(s): YYYY-MM-DD

Reporting Summary

Nature Research wishes to improve the reproducibility of the work that we publish. This form provides structure for consistency and transparency in reporting. For further information on Nature Research policies, see Authors & Referees and the Editorial Policy Checklist.

Statistics

For all statistical analyses, confirm that the following items are present in the figure legend, table legend, main text, or Methods section.

n/a	Confirmed	
☐	☒	The exact sample size (*n*) for each experimental group/condition, given as a discrete number and unit of measurement
☐	☒	A statement on whether measurements were taken from distinct samples or whether the same sample was measured repeatedly
☐	☒	The statistical test(s) used AND whether they are one- or two-sided<br>*Only common tests should be described solely by name; describe more complex techniques in the Methods section.*
☐	☒	A description of all covariates tested
☐	☒	A description of any assumptions or corrections, such as tests of normality and adjustment for multiple comparisons
☐	☒	A full description of the statistical parameters including central tendency (e.g. means) or other basic estimates (e.g. regression coefficient) AND variation (e.g. standard deviation) or associated estimates of uncertainty (e.g. confidence intervals)
☐	☒	For null hypothesis testing, the test statistic (e.g. *F*, *t*, *r*) with confidence intervals, effect sizes, degrees of freedom and *P* value noted<br>*Give P values as exact values whenever suitable.*
☒	☐	For Bayesian analysis, information on the choice of priors and Markov chain Monte Carlo settings
☒	☐	For hierarchical and complex designs, identification of the appropriate level for tests and full reporting of outcomes
☐	☒	Estimates of effect sizes (e.g. Cohen's *d*, Pearson's *r*), indicating how they were calculated

Our web collection on statistics for biologists contains articles on many of the points above.

Software and code

Policy information about availability of computer code

Data collection	Common Mind Consortium
Data analysis	Custom code

For manuscripts utilizing custom algorithms or software that are central to the research but not yet described in published literature, software must be made available to editors/reviewers. We strongly encourage code deposition in a community repository (e.g. GitHub). See the Nature Research guidelines for submitting code & software for further information.

Data

Policy information about availability of data

All manuscripts must include a data availability statement. This statement should provide the following information, where applicable:

- Accession codes, unique identifiers, or web links for publicly available datasets
- A list of figures that have associated raw data
- A description of any restrictions on data availability

Data used in this study are available through the CommonMind Consortium: CommonMindConsortiumDataAccess@synapse.org

Field-specific reporting

Please select the one below that is the best fit for your research. If you are not sure, read the appropriate sections before making your selection.

☒ Life sciences ☐ Behavioural & social sciences ☐ Ecological, evolutionary & environmental sciences

Life sciences study design

All studies must disclose on these points even when the disclosure is negative.

Sample size	Sample size was determined based on availability of postmortem brain samples: subjects with known time of death (TOD) and meeting the criteria of rapid death (< 2 hours elapsed time between precipitating event and death pronouncement); 2) subjects with age less than 65 years; 3) subjects with post-mortem interval (PMI) less than 30 hours. 150 subjects fit these criteria, including 104 healthy control subjects and 46 subjects with schizophrenia (SCZ). To account for sample size differences between the healthy control and SCZ cohorts, when comparing these two groups, we used a subset of the healthy control subjects with the same sample size as the SCZ cohort (N = 46).
Data exclusions	We did not exclude any data.
Replication	As shown in Supplementary Figure 1, we showed reproducibility of our rhythmic analysis in healthy control subjects. We compared the phase of the top rhythmic genes between the current dataset (RNA-seq data) and our previous study (microarray data) from another cortical brain region. With respect to our finding that differential expression in SCZ vs. healthy control subjects during the night drives differential expression when day and night subjects are combined, we compared our differential expression at night findings to a previously published dataset (Gandal et al., Science, 2018). We find a statistically significant overlap in these two gene lists, supporting the replicability of our findings.
Randomization	For the full healthy control cohort, we used all subjects that fulfilled our time of death criteria. For the matched healthy control and schizophrenia (SCZ) cohort, we selected healthy control subjects such that group parameters did not differ between groups (Supplementary Table 5). Specifically, these groups did not differ statistically in proportion of males and females, racial make-up, age, postmortem interval, brain pH, time of death, and proportion of subjects from the University of Pittsburgh or Mount Sinai brain banks.
Blinding	Data used in this study were previously generated by the CommonMind Consortium.

Reporting for specific materials, systems and methods

We require information from authors about some types of materials, experimental systems and methods used in many studies. Here, indicate whether each material, system or method listed is relevant to your study. If you are not sure if a list item applies to your research, read the appropriate section before selecting a response.

Materials & experimental systems

n/a	Involved in the study
☒	☐ Antibodies
☒	☐ Eukaryotic cell lines
☒	☐ Palaeontology
☒	☐ Animals and other organisms
☒	☐ Human research participants
☒	☐ Clinical data

Methods

n/a	Involved in the study
☒	☐ ChIP-seq
☒	☐ Flow cytometry
☒	☐ MRI-based neuroimaging
